# Supplementary material for: Temporal Trends in Renal Replacement Therapy in Community-Based People with or without Type 2 Diabetes: The Fremantle Diabetes Study
Source: J Clin Med. 2022 Jan 28;11(3):695. doi: 10.3390/jcm11030695 (PMC8837160; doi:10.3390/jcm11030695)
Supplement: Supplementary file 1 [file jcm-11-00695-s001.zip › jcm-1525723-supplementary.pdf]

Table S1: Summary of imputation of data in analyses.

Variable	No. (%) missing cases	In multiple imputation model	Imputed	In final risk models
Age	0	Y	N	N
Sex	0	Y	N	N
Ethnic background	0	Y	N	Y
Coronary heart disease before study entry	0	Y	N	N
Myocardial infarction before study entry	0	Y	N	N
Heart failure before study entry	0	Y	N	N
Lower extremity amputation before study entry	0	Y	N	N
End-stage renal failure before study entry	0	Y	N	N
Charlson Comorbidity Index in 5 years before study entry	0	Y	N	N
All-cause death within 5 years of study entry	4 (0.1)	Y	N	N
Cardiovascular disease death within 5 years of study entry	9 (0.3)	Y	N	N
Myocardial infarction within 5 years of study entry	8 (0.3)	Y	N	N
Stroke within 5 years of study entry	9 (0.3)	Y	N	N
Heart failure within 5 years of study entry	4 (0.1)	Y	N	N
Lower extremity amputation within 5 years of study entry	4 (0.1)	Y	N	N
End-stage renal disease within 5 years of study entry	4 (0.1)	Y	N	Y (outcome)
Marital status	3 (0.1)	Y	Y	N

Current smoker	22 (0.8)	Y	Y	N
Height	6 (0.2)	Y	Y	N
BMI	11 (0.4)	Y	Y	N
Heart rate	12 (0.4)	Y	Y	N
Diastolic blood pressure	6 (0.2)	Y	Y	N
Antihypertensive medication	5 (0.2)	Y	Y	N
Age at diabetes diagnosis	8 (0.3)	Y	Y	N
HbA_{1c}	16 (0.6)	Y	Y	N
On insulin	7 (0.2)	Y	Y	N
Total serum cholesterol	18 (0.6)	Y	Y	N
HDL-cholesterol	23 (0.8)	Y	Y	N
Serum triglycerides	18 (0.6)	Y	Y	N
Lipid-modifying medication	9 (0.3)	Y	Y	N
Aspirin use	5 (0.2)	Y	Y	N
Urinary albumin:creatinine ratio	63 (2.2)	Y	Y	Y
eGFR	19 (0.7)	Y	Y	Y
Baseline foot ulcer	9 (0.3)	Y	Y	N
Baseline foot pulse	19 (0.7)	Y	Y	N
Peripheral sensory neuropathy	66 (2.4)	Y	Y	N
Peripheral arterial disease	32 (1.1)	Y	Y	N
Atrial fibrillation	45 (1.6)	Y	Y	N

Table S2: Characteristics at study entry of type 2 diabetes participants in FDS1 and FDS2 combined by 5-year incident renal replacement therapy (RRT) in those with no prior hospitalization for/with RRT.

	No RRT	RRT	*P*-value
Number (%)	2769 (99.20)	22 (0.8)	
In FDS2 (%)	53.4	77.3	0.031
Time from start of Phase to participant entry (years)	1.41±0.90	1.81±0.93	0.036
Age at FDS entry (years)	64.8±11.5	59.3±15.0	0.10
Sex (% male)	50.4	40.9	0.40
Overseas born (%)	45.2	45.5	>0.99
Aboriginal (%)	4.0	36.4	<0.001
Education beyond primary level (%)	80.8	95.2	0.16
Currently married/de facto (%)	64.1	59.1	0.66
Alcohol (standard drinks/day)	0.1 [0-0.8]	0.1 [0-0.8]	0.72
Ever smoker (%)	54.9	59.1	0.83
Age at diagnosis (years)	56.8±12.0	43.6±15.7	<0.001
Duration of diabetes (years)	5.0 [1.8-12.9]	15.2 [8.5-22.2]	0.001
Insulin use ± oral agents (%)	17.2	36.4	0.040
Fasting glucose (mmol/L)	7.5 [6.2-9.6]	6.4 [5.5-8.4]	0.08
HbA_{1c} (%)	7.0 [6.2-8.1]	7.4 [6.2-8.7]	0.50
Self-reported hypoglycemia last year (%):	28.6	54.5	0.015
BMI (kg/m^2)	30.5±5.9	31.8±7.7	0.28
Obesity (% by waist circumference)	68.0	77.3	0.49
SBP (mm Hg)	148±23	165±35	0.028
DBP (mm Hg)	80±12	86±17	0.13

BP-lowering medication (%)	62.8	68.2	0.66
Heart rate (bpm)	70±12	72±12	0.41
Total cholesterol (mmol/L)	4.9±1.2	5.4±2.1	0.24
HDL-cholesterol (mmol/L)	1.15±0.35	1.12±0.41	0.62
Serum triglycerides (mmol/L)	1.8 (1.0-3.2)	2.5 (1.5-4.1)	0.007
Lipid-modifying medication (%)	41.3	50.0	0.52
Aspirin use (%)	30.2	≤22.7*	0.25
Serum uric acid (µmol/l)	0.36±0.10	0.47±0.11	<0.001
Urinary albumin:creatinine (mg/mmol)	3.9 (1.1-13.8)	210.0 (45.5-969.3)	<0.001
eGFR (CKD-EPI) categories (%):			<0.001
≥60 ml/min/1.73m^2	83.7	14.3	
30-59 ml/min/1.73m^2	14.8	33.3	
15-29 ml/min/1.73m^2	1.3	28.6	
<30 ml/min/1.73m^2	0.1	23.8	
Atrial fibrillation (%)	4.6	18.2	0.017
Hospitalization for/with heart failure (%)	7.0	27.3	0.003
Hospitalization for/with myocardial infarction (%)	8.3	≤22.7*	0.71
Ischemic heart disease (%)	29.1	≤22.7*	0.35
Hospitalization for/with stroke (%)	1.7	≤22.7*	>0.99
Cerebrovascular disease (%)	10.4	≤22.7*	>0.99
Hospitalization for lower extremity amputation (%)	1.0	≤22.7*	0.21
Peripheral arterial disease (%)	25.7	≤22.7*	0.23
Peripheral sensory neuropathy (%)	45.6	54.5	0.52
Depressive symptoms (%)	27.0	41.2	0.27
ApoE4 allele (%)	22.8	19.0	0.80
Charlson Comorbidity Index[a] (%):			0.001
0	74.1	45.5	

	1-2	19.1	27.3
	≥3	6.8	27.3

*actual percentages not given to preserve confidentiality; [a]in the last 5 years, excluding diabetes and its complications
